# Hemorheological Alterations and Oxidative Damage in Sickle Cell Anemia

**DOI:** 10.3389/fmolb.2019.00142

**Published:** 2019-12-04

**Authors:** Patrizia Caprari, Sara Massimi, Loretta Diana, Francesco Sorrentino, Laura Maffei, Stefano Materazzi, Roberta Risoluti

**Affiliations:** ^1^National Centre for the Control and Evaluation of Medicine, IstitutoSuperiore di Sanità, Rome, Italy; ^2^Thalassemia Unit, S. Eugenio Hospital, Rome, Italy; ^3^Department of Chemistry, Sapienza University of Rome, Rome, Italy

**Keywords:** sickle cell anemia, hemorheology, oxidative damage, erythrocyte deformability, erythrocyte membrane

## Abstract

Sickle cell anemia (SCA) is the most common hereditary disorder of hemoglobin (Hb) characterized by a mutation in the β globin gene, which leads to synthesis of HbS a hemoglobin which, under hypoxic conditions, gels and leading to the sickling of the red blood cells (RBC). The dehydration of the RBC increases the concentration of the intracellular Hb with an increase in the internal viscosity and consequently a decrease in the erythrocyte deformability. Sickle red blood cells due to their difficulty to flow through the microcirculation cause frequent vaso-occlusive episodes, tissue ischemia, and infarctions. Moreover, the reduced RBC deformability causes cell fragility leading to hemolysis and recently a key role of hemolysis and oxidative stress in the development of vascular dysfunction has been demonstrated. The aim of this study was to evaluate the hemorheological profiles of patients with SCA in order to point out new indices of vascular impairment, and to characterize the membrane oxidative damage of sickled RBC. Blood viscosities, erythrocyte aggregation, and viscoelastic profiles of SCA patients were determined, and the RBC oxidative damage was investigated by comparing metabolic capability and RBC membrane proteins from SCA patients with and without transfusion dependence. The hemorheological profile of SCA subjects demonstrated high blood viscosity, increased RBC aggregation, and decreased RBC deformability. These impaired flow properties were associated with RBC membrane protein oxidation, with degradation of spectrin and increased membrane-bound globin. The comparison between SCA patients with and without transfusion dependence showed metabolic and structural RBC oxidative damage significantly different.

## Introduction

Sickle cell anemia (SCA) is the most common hereditary disorder of hemoglobin synthesis characterized by a mutation in the β globin gene, which leads to the replacement of glutamic acid with valine at the sixth codon and synthesis of Hb S a hemoglobin which, under hypoxic conditions, gels leading to the sickening of the red blood cells (RBC). The dehydration of the RBC increases the concentration of the intracellular Hb with an increase in the internal viscosity and consequently a decrease in the erythrocyte deformability (Ballas and Mohandas, 2004; Rees et al., 2010; Azar and Wong, 2017; Ware et al., 2017).

Blood viscosity and erythrocyte deformability are the main determinants the maintenance and regulation of microcirculation. Hemorheological changes produced by alterations both of blood cells and plasma components induce a rise in blood viscosity, which may slow blood flow and cause occlusions through the erythrocyte rouleaux formation and the platelet aggregation. Sickle red blood cells due to their difficulty to flow through the microcirculation, cause frequent vaso-occlusive episodes, poor microvascular blood flow, tissue ischemia, and infarction (Bowers et al., 2013, 2018; Connes et al., 2016; Azar and Wong, 2017; Risoluti et al., 2017; Ware et al., 2017). Moreover, the reduced RBC deformability causes an increased cell fragility leading to enhanced hemolysis (Grau et al., 2013) and a key role of hemolysis and oxidative stress in the development of vascular dysfunction has been demonstrated (Barodka et al., 2014; Connes et al., 2014; Hierso et al., 2014; Hermann et al., 2016; Mockesch et al., 2017; Renoux et al., 2018). A reduction in RBC deformability associated with an increase in oxidative stress has been observed in several pathological conditions such as hypertension and diabetes (Lee et al., 2017; Diederich et al., 2018). In patients with retinal vein occlusion, hemorheological alterations associated with the erythrocyte oxidative stress and consisting in increased blood viscosity and decreased RBC deformability have also been described (Becatti et al., 2016). These studies have been confirmed by *in vitro* experiments demonstrating that reactive oxygen species modify the fluidity of the erythrocyte membrane (Becatti et al., 2017; Diederich et al., 2018).

Sickle RBCs and their membranes are sensitive to auto-oxidation due to endogenous activated oxygen species (Lux et al., 1976; Rice-Evans and Omorphos, 1983; Platt et al., 1985; Rice-Evans et al., 1986; Amer et al., 2006). *In vitro* studies on the exposure of sickle cells to oxidative stress with different oxidants (t-butylhydroperoxide, hydrazine, diamide, hydrogen peroxide) have demonstrated lipid peroxidation and irreversible hemoglobin denaturation more pronounced in sickle RBCs than normal ones (Snyder et al., 1981; Hebbel et al., 1982; Rice-Evans and Baysal, 1987). Moreover, in sickle cell disease a reduced capability to counteract the oxidation was described, and the effects of antioxidants, free radical scavengers, and also iron chelators on sickle RBCs and their membranes have been evaluated (Chiu and Lubin, 1979; Das and Nair, 1980; Rice-Evans et al., 1986; Rice-Evans and Baysal, 1987; Amer et al., 2006; Gizi et al., 2011; Voskou et al., 2015).

The aim of this study was to evaluate the hemorheological profiles of patients with SCA in order to point out new indices of vascular impairment, and to characterize the membrane oxidative damage of sickled RBC from patients with and without transfusion therapy dependence. The whole blood viscosity, plasma viscosity, erythrocyte aggregation index, and blood viscoelastic profiles of SCA patients, in comparison with that of healthy subjects, have been determined. Furthermore, the oxidative membrane damage of RBC in SCA have been investigated by comparing metabolic capability and membrane structure integrity of RBC from transfusion dependent (TD-SCA) and non-transfusion-dependent (NTD-SCA) patients. The hemorheological profile of SCA subjects demonstrated high blood viscosity at both low and high shear rates, increased RBC aggregation, and decreased RBC deformability. These impaired flow properties were associated with metabolic and structural RBC oxidative damage significantly different in TD-SCA and NTD-SCA patients.

## Methods

### Subjects

In this study the analysis of blood samples from subjects affected by SCA and healthy individuals was performed. Blood collecting protocol and all experimental procedures were approved by the local ethics committee and the patients gave their written informed consentto participate in this study, which conforms to the International Compilation of Human Research Standards (Department of Health and Human Services, 2011). The study was done in accordance with the principles of Good Clinical Practice, the Declaration of Helsinki, and all the local regulations.

### Hematological and Biochemical Analyses

Blood samples collected on K2-EDTA were analized within 2–3 h. Red blood cell indices were determined by ADVIA 120 (Siemens, USA). Hemoglobins quantification was performed by high-pressure liquid chromatography (HPLC-Variant, Bio-Rad). Erythrocyte enzyme activities pyruvate kinase (PK) and glucose-6-phosphate dehydrogenase were assayed according to the International Committee for Standardization in Hematology (Beutler et al., 1977).

The reduced glutathione (GSH) content was determined by assessing the reduction of 5,5′-dithiobis(2-nitrobenzoic acid) (DTNB) by sulfhydryl compounds at λ 412 nm (Beutler, 1971). The GSH content was expressed as μmol/gHb.

Adenosine triphosphate (ATP) concentration was determined on whole blood perchloric acid extract by an enzyme assay at 340 nm at 37°C according to International Committee for Standardization in Haematology (ICSH) (1989) methods. The ATP concentration was expressed as μmol/gHb.

### Membrane Protein Analysis

Leukocyte and platelet free red cells were obtained by filtration through microcrystalline cellulose-α-cellulose (1:1) column equilibrated with a phosphate-buffered saline (5 mM Na/Na_2_HPO_4_, pH 7.4, 0.15 M NaCl, 0.1 mM PMSF). The erythrocytes were washed twice with the same buffer, and lysed with hypotonic buffer (5 mM Na_2_HpO_4_ pH 8.0, 0.1 mM PMSF) in 1:30 (v/v) ratio to obtain erythrocyte membrane. The ghosts were washed three times with the same buffer (Caprari et al., 1995). RBC membrane proteins were analyzed by 7.5% polyacrylamide gel electrophoresis in sodium dodecyl sulfate (SDS-PAGE) according to Laemmli (1970) with slight modifications (Caprari et al., 1991). RBC ghosts were dissolved in SDS sample buffer (2% SDS, 5% 2-mercaptoethanol, 10% glycerol, 0.001% bromophenol blue, 63 mM Tri-HCl, pH 6.8) to a concentration of 1 mg/ml, and, after an incubation for 2 min at 95°C, loaded on the gel. Staining of protein bands was performed with Coomassie Blue and a laser beam densitometer (Gel Doc XR+) equipped with a Lab Image Software Package (BIORAD) was use to quantify the percentage of the membrane proteins.

### Hemorheological Assays

Hemorheological profile analysis was carried out by Rheo-Microscope (Anton Paar, Germany) that is a glass parallel platerheometer, Physica MCR301, with a Peltier system for temperature control (37 ± 0.5°C). Whole Blood Viscosity (WBV) (η) was determined at shear rates 1 s^−1^ (η1) and 200 s^−1^ (η200), low and high shear rates, respectively, according to the Recommendation of the International Committee for Standardization in Haematology (ICSH) (1986) and the International Expert Panel for Standardization of Hemorheological (Baskurt et al., 2009). Plasma viscosity (ηpl) testing was performed at shear rates 200 s^−1^. Erythrocyte Aggregation Index (EAI) was determined as η1 and η200 ratio (η1/η200). Since blood viscosity increases with the rise of hematocrit, these hemorheological parameters were determined in conditions of native and normalized hematocrit (i.e., adjusted to 40 or 45%, for females and males, respectively). RBCs viscoelastic properties were evaluated by determining elastic modulus G′, viscous modulus G″, and the tangent of phase shift angle (Tgδ = G″/G′) as a function of strain rate at a constant value of deformation amplitude in the range from 0.1 to 10 Hz (*f* = ω/2π). The values of the modules G′ and G″, and Tgδ, expressed in Pa, were determined by oscillatory measurements in the range of viscoelastic linearity (10% deformation), as previously described (Martorana et al., 2007).

## Results

### Patients

Twenty-four patients affected by SCA and followed by the Thalassemia Unit of S. Eugenio Hospital of Rome were enrolled for this study between 2016 and 2018. The protocol for diagnosis of SCA included an assessment of the patient's clinical presentation together with screening tests, and molecular characterization of globin genes mutations (Materazzi et al., 2014, 2017a; Green et al., 2015; Risoluti et al., 2016, 2018; Catauro et al., 2018).

In Table 1 are shown clinical and demographic characterization of the patients. Fourteen SCA patients (age 35 ± 14 years, mean ± standard deviation) were transfusion dependent (TD-SCA) and the age of SCA diagnosis ranged was from 1 to 18 years. In this group (7 males/7 females) two women were of African origin, a man was from Albania and the others patients had Italian origin. There were n. 4 subjects with HbS homozygosity, n. 1 with double heterozygosity for HbS/HbD, n. 7 with heterozygosity for HbS/β-thalassemia (HbS/β-thal), and n. 2 subjects with HbS/β-thal/α 3.7-thal.

**Table 1:** Clinical and hematological characterization of SCA patients: comparison between transfusion-dependent (TD) and non-transfusion-dependent (NTD) subjects.

**Patients**	**TD**	**NTD**
*N*	14	10
Age	35 ± 14	30 ± 10
Gender	7 M/7 F	6 M/4 F
Origin country (*n*)	Italy (11) Africa (2) Albania (1)	Italy (8) Africa (2)
Genotype (*n*)	Hb S/Hb S (4) Hb S/Hb D (1) HbS/β Thal (7) HbS/β Thal/α-3.7 (2)	Hb S/Hb S (2) HbS/β Thal (7) HbS/β Thal/α-3.7 (1)
Clinical phenotype	n. 6 severe n. 8 mild	n. 2 severe n. 8 mild
Complications	Pretibial ulcers, bone pains, bone infarcts Aseptic necrosis of femora Transient Ischemic Attack (TIA)	Aseptic necrosis of femora Portal carvenoma Bone infarcts
Hb S (%)	29 ± 8	70 ± 5
Hb F (%)	5.0 ± 3.2	14.1 ± 12.5
Hb (g/dL)	10.9 ± 1.5	10.6 ± 2.0
Hct (%)	33.0 ± 4.4	32.8 ± 5.9
RDW (%)	19.0 ± 2.6	19.5 ± 3.1
PLT (10^9^/L)	399 ± 142	308 ± 165
Ret (10^9^/L)	268 ± 124	237 ± 97

*The values are expressed as mean ± standard deviation*.

Ten SCA patients (age 30 ± 10 years, mean ± standard deviation) were non-trans fusion dependent (NTD-SCA), and the age of SCA diagnosis ranged was from 2 to 32 years. In the NTD group (6 males/4 females) there were n. 2 women of African origin with genotype HbSS, and the other eight patients of Italian origin with genotypes heterozygotes for HbS/β-thal (7 subjects), and a heterozygote for HbS/β-thal/α 3.7-thal. All the patients did not had been splenectomized, and the TD patients had a median free time to transfusion of 30 days, and have been analyzed far from transfusion.

Thirty healthy subjects (18 males/12 females) of Italian origin with age 30 ± 10 years (mean ± standard deviation) were used as controls (CTR).

### Clinical Data

The SCA patients showed a wide heterogeneity concerning the severity of clinical symptoms and complications (Table 1). Generally, TD patients showed a severe clinical phenotype associated with typical symptoms and signs of SCA: bone infarcts, pretibial ulcers, aseptic necrosis of femora, and transient ischemic attach (TIA), also recurrent. The clinical phenotype of the NTD patients was milder with the exception of two patients (Table 1) and the complications observed were avascular necrosis of the femoral head, portal cavernoma, and in few cases bone infarcts.

### Hematological Data

The hematological data (Table 1) showed reduced contents of HbS (29 ± 8%) and Hb F (5.0 ± 3.2%) in TD patients as the effect of transfusion, while the NTD patients showed significantly high values of HbS (70 ± 5%) (*p* < 0.0001) and variable values of HbF (14.1 ± 12.5%). Comparable values of Hb and hematocrit (Hct), and high values of red cell distribution width (RDW) and reticulocyte counts were determined in TD and NTD SCA patients.

### Hemorheological Profile

**Figure 1:**
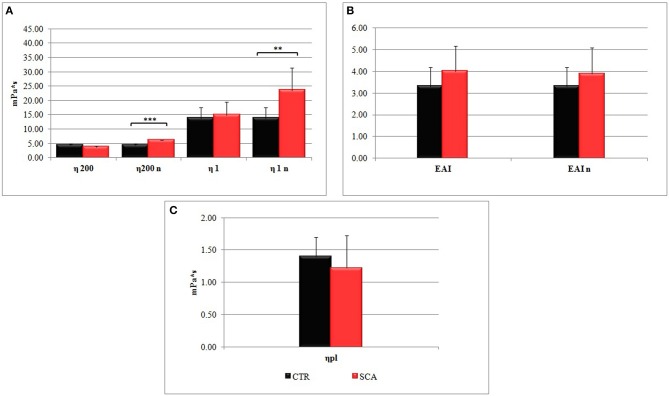
Hemorheological profiles of sickle cell anemia (SCA) patients and healthy subjects (SCA): **(A)** whole blood viscosities η200 and η1 determined at low shear rates (1 s^−1^) and high shear rates (200 s^−1^), and **(B)** the erythrocyte aggregation index (EIA) (η1/η200) in conditions of native hematocrit. The values η200n, η1n, and EIAn were obtained at normalized hematocrit (Hct 40–45%). **(C)** Plasma viscosity (ηpl) is determined at shear rates 200 s^−1^.

The hemorheological profile of SCA patients was compared with that of healthy subjects. We have analyzed the whole blood viscosities (η) determined at low (η1) and high (η200) shear rates, and the erythrocyte aggregation index in conditions of both native and normalized hematocrit (Figure 1).

The values η200n, and η1n of SCA patients obtained at normalized hematocrit (Hct 40–45%) were significantly higher than control ones (Figure 1A), particularly the values of η1n, which are indicative of a high viscosity at low shear rates, which correspond to the microcirculation. In agreement with this observation the values EAI of SCA patients (Figure 1B) were higher than that of healthy subjects. Plasma viscosity resulted lower in SCA patients than in control subjects (Figure 1C).

Viscoelastic properties were analyzed by determining the storage modulus G′ (elastic modulus), the loss modulus G″ (viscous modulus), and the tangent δ from SCA patients and healthy subjects.

**Figure 2:**
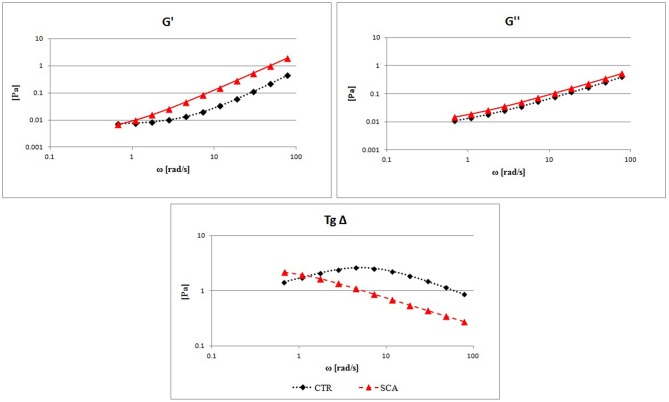
Evaluation of RBCs viscoelastic properties from sickle cell anemia (SCA) patients and healthy subjects (CTR): elastic modulus G′, viscous modulus G″, and the tangent of phase shift angle (Tgδ = G″/G′) as a function of strain rate at a constant value of deformation amplitude, selected on the linear viscoelastic range by strain test.

In Figure 2 the comparison between the G′, G″, and Tgδ mean curves are showed. High values of G′ are found in SCA patients in comparison with the control ones demonstrating high rigidity of the RBC and decreased deformability, associated with a constant increase in G″ which represents an increase in viscous modulus, in agreement with the previously reported blood viscosity results. The tangent δ curve, which represents the combination of the two components G′ and G″, shows a highly altered viscoelastic profile in patients with SCA compared to that of healthy subjects. The tangent δ curve is far below the normal curve to indicate a severe reduction of erythrocyte deformability with increase rigidity of the RBC membrane.

### Oxidative Damage of RBC Membrane

The oxidative damage of RBC membrane was investigated by comparing both membrane structure integrity and metabolic capability of RBC from healthy subjects and SCA patients divided in TD and NTD, since periodic transfusion of normal red blood cells may be an additional factor influencing oxidative stress in SCA. As markers of the RBC metabolic and reduction capabilities we have chosen the metabolic intermediate GSH and ATP and the related enzyme activities G6PD and PK (Table 2).

**Table 2:** Oxidative damage of RBCs from transfusion-dependent SCA patients (TD-SCA), not transfusion-dependent SCA patients (NTD-SCA), and healthy subjects (CTR).

	**TD**	**NTD**	**CTR**	**TD vs. NTD**	**CTR vs. TD**	**CTR vs. NTD**
GSH (μmol/gHb)	7.4 ± 0.4	6.7 ± 0.8	7.2 ± 0.6	*p* = 0.01		
G6PD (IU/gHb)	16.3 ± 5.1	21.3 ± 4.8	11.5 ± 2.6	*p* < 0.05	*p* < 0.05	*p* < 0.0001
ATP (μmol/gHb)	5.0 ± 0.7	4.8 ± 0.9	4.1 ± 0.4		*p* < 0.01	
PK (IU/gHb)	25.9 ± 7.5	34.2 ± 3.6	15.2 ± 2.7	*p* < 0.05	*p* = 0.001	*p* < 0.0001
Spectrin (%)	24.5 ± 2.8	30.3 ± 5.4	30.9 ± 3.3	*p* < 0.05	*p* < 0.05	
4.1 b (%)	1.6 ± 0.5	1.8 ± 0.5	1.3 ± 0.3			*p* < 0.05
Globin (%)	9.1 ± 3.4	4.1 ± 3.3	1.8 ± 2.0	*p* < 0.01	*p* < 0.01	

*The oxidative membrane damage was investigated by comparing metabolic capability and membrane structure integrity of RBC from SCA patients with and without transfusion dependence. RBCs, red blood cells; GSH, reduced glutathione; ATP, adenosine triphosphate; G6PD, glucose-6-phosphate dehydrogenase activity; PK, pyruvate kinase activity. The values are expressed as mean ± standard deviation. The statistical evaluation between the groups was performed by t Student test*.

Significantly high values of G6PD and PK were found in both TD and NTD patients as compared with control ones, and significantly differences were observed also between the two groups of patients with the highest values in NTD patients. These high values of G6PD and PK are closely related to the high reticulocyte counts in SCA patients, confirm the presence of a hemolytic state, and are indicative of a greater request of metabolic and reducing capacities for erythrocyte survival to which the RBCs respond with production of ATP and GSH. Indeed, ATP values were higher in SCA patients in comparison with the controls' ones and this difference was significant (*P* < 0.01) between TD and CTR. Erythrocyte GSH values were within normal values, but significant differences were observed between TD and NTD patients (*p* = 0.001) with GSH mean value of NTD-SCA subjects lower in comparison with the values of TD-SCA and controls.

The analysis of the erythrocyte membrane proteins demonstrated an impairment of the eryhtrocyte membrane of TD-SCA patients since spectrin content was significantly lower than spectrin contents of both NTD-SCA patients and healthy subjects (*p* < 0.05). NTD-SCA patients had a normal value of membrane spectrin. Moreover, the electrophoretic patterns of membrane showed an evident band of globin bound to the membrane in SCA patients in comparison with controls. The membrane globin content was significantly higher in TD-SCA in comparison with values of NTD-SCA and healthy subjects. The concentrations of the membrane proteins ankyrin, protein B and 3, protein 4.1, and protein 4.2 were within the normal values and comparable between SCA patients and healthy subjects. It is also noteworthy an increase in the protein 4.1b content of the membrane in the RBCs of SCA patients, this increase reaches significant differences in NTD patients compared to healthy subjects.

## Discussion

The clinical manifestations of SCA are extremely heterogeneous from asymptomatic individuals to patients with severe and recurrent pain crisis, ischemia, inflammatory state, and chronic complications. In the homozygous (HbSS) and in double heterozygous (HbS/β thalassemia) conditions four major morbidities are frequently observed: chronic hemolytic anemia, systemic manifestations with susceptibility to infections, painful vaso-occlusive crises (VOC) that can vary from patient to patient in intensity and frequency, and multi-organ damage.

Generally patients are anemic and from the hemorheological point of view (Connes et al., 2016) the low hematocrit value can in part compensate the sickle cell rigidity and determine a blood viscosity in the normal ranges, as we observed in the patients analyzed in this study. However, when the hematocrit increases, even toward values considered in the norm, an increase in blood viscosity may extremely relevant and determine, in conditions of slow flow as in the microcirculation, an increased erythrocyte aggregation. The results of this study show alterations in blood viscosity in hematocrit conditions at 40–45% that concern both the viscosity at low shear rates and the erythrocyte aggregation index, and the viscosity at high shear rates representing the macrocirculation. Therefore, the evaluation of blood viscosity and erythrocyte aggregation should be carried out both with native hematocrit and normalized hematocrit to be able to evaluate the effect in conditions of constrictions and vaso-occlusions in the microcirculation.

The study of the viscoelastic profile of SCA subjects demonstrated high rigidity of the RBC and decreased deformability, associated with a constant increase in G″ which represents an increase in viscous modulus, in agreement with the blood viscosity results. The tangent δ curve of RBC from SCA patients is far below the normal curve to indicate a severe reduction of erythrocyte deformability with increase rigidity of the RBC membrane. Overall these results confirm that red blood cells of SCA patients have impaired flow properties with enhanced aggregability, and reduced deformability that induce micro-circulatory disorders. These hemorheological alterations can derive from the structural alterations of the red blood cell induced by oxidative stress. Several recent studies have investigated the role of oxidative stress in SCA and in the progression of complications (Agas et al., 2008; Barodka et al., 2014; Grau et al., 2015; Materazzi et al., 2017b; Mockesch et al., 2017; Biswal et al., 2018; Renoux et al., 2018). The studies have described several causes of oxidative stress in SCA, including NADPH-oxidase-derived superoxide from endothelial cells, from activated poly-morphonuclear neutrophils, as well as from RBCs. Moreover, the free hemoglobin and hem into plasma are known to induce the formation of reactive oxygen species (ROS) and reactive nitrogen species (RNS), with a decrease in nitric oxide. ROS and RNS are also produced inside the sickle RBCs for the HbS auto-oxidation in the cycles of sickling/unsickling (Hierso et al., 2014).

The important role that membrane proteins play in the ability to deform red blood cells is known and congenital alterations of these proteins can cause hemolysis of erythrocytes and anemia such as hereditary spherocytosis, and hereditary elliptocytosis. Several studies on the effect of *in vitro* oxidative stress on RBC demonstrated alteration of the skeletal network at the horizontal junction sites involving spectrin, actin, and protein 4.1 thus to modify the cytoskeletal assembly, and to play a role in the hemolytic process (Lux et al., 1976; Platt et al., 1985; Rice-Evans et al., 1986; Caprari et al., 1995; Rees et al., 2010; Gizi et al., 2011). The studies reported in the literature do not demonstrate quantitative defects of the main membrane and cytoskeletal proteins in SCA (Lux et al., 1976; Schwartz et al., 1987), but alterations in the interactions between the main components: spectrin, ankyrin, and protein 4.1. It should be noted, that the patients analyzed are very heterogeneous, some authors have only studied patients with HbS homozygosity, while others have studied patients with both homozygous and heterozygous SCA. To our knowledge, there are no studies that have described the oxidative damage of membrane proteins by comparing SCA patients with and without transfusion dependence. Blood transfusions are life-saving therapy for many patients with SCA, mainly patients with severe SCA forms. In this study we have demonstrated that a degradation of spectrin in addition to the increase in membrane-bound globin are the relevant alterations of membrane in SCA producing reduced RBC viscoelastic properties leading both to a reduced RBC deformability and probably clustering of Band 3, as previously reported (Hierso et al., 2014). The increased content of protein 4.1b, the high values of PK and G6PD activities and the high reticolocyte counts in SCA are indicative of a reduced mean age of RBC population caused by to hemolysis. To a greater request of antioxidant capacity the RBCs respond with production of ATP and GSH, both in TD and in NTD patients, with ATP values higher in SCA patients in comparison with the controls' ones. The comparison between SCA patients with and without transfusion dependence showed metabolic and structural oxidative damage of RBC significantly different, that could be attributable in part to the RBCs for transfusion. Some studies reported GSH values lower in SCA RBCs as compared to healthy individuals, as well as impaired catalase activity and other proteins involved in antioxidant protection (Amer et al., 2006; Voskou et al., 2015). On the contrary, as concerns the concentrations of ATP and GSH, and G6PD activity, our results are in agreement with the study of Rice-Evans et al. (1986) on homozygous SCA patients in which they found that the oxidative damage correlated with the proportion of irreversibly sickled cells. Comparable GSH content, increased G6PD activity, and similar ATP content were determined in SCA patients with low sickle cells, and a decrease in ATP values only in SCA patients with 5–25% sickle cells was observed. This observation highlights the importance of transfusion therapy to balance the oxidative damage.

In conclusion RBC physiology is severely impaired in SCA, and this contributes to the chronic vascular dysfunction. Many factors affect both clinical severity and frequency of VOC episodes. In addition to the globin genes genotype, and HbS and HbF content, also the alterations of the hemorhelogical profile and oxidation of the RBC membrane proteins with degradation of spectrin and membrane-bound globin are relevant. The determination of the hemorheological parameters and the evaluation of the oxidative damage of the membrane may result important for a better understanding the heterogeneity of clinical signs and the pathophysiology of SCA. The introduction of these new parameters of evaluation could give useful information to carry out personalized therapeutic and care protocols in SCA patients.

## Data Availability Statement

The datasets generated for this study are available on request to the corresponding author.

## Ethics Statement

The studies involving human participants were reviewed and approved by Comitato Etico Roma 2 of the S. Eugenio Hospital, Rome. Written informed consent for participation in the study and publication of clinical data in an anonymized manner were collected and copies of the informed consent are available on request.

## Author Contributions

PC, RR, and SMat conceived and designed the study, wrote the manuscript, and evaluated data for statistics. FS and LM enrolled the patients, performed the clinical evaluation, and management of subjects. SMas and LD performed experiments. All the authors have revised and approved the final version of the manuscript.

### Conflict of Interest

The authors declare that the research was conducted in the absence of any commercial or financial relationships that could be construed as a potential conflict of interest.
